# Psychiatric Co‐Morbidities Among Children and Adolescents With Headache: Findings From a Cross‐Sectional Study in Bangladesh

**DOI:** 10.1002/brb3.70599

**Published:** 2025-05-30

**Authors:** Sifat E. Syed, Mohammad S. I. Mullick

**Affiliations:** ^1^ Department of Psychiatry Bangabandhu Sheikh Mujib Medical University (BSMMU) Dhaka Bangladesh

**Keywords:** Bangladesh, children, pediatric headache, psychiatric comorbidity

## Abstract

**Introduction:**

Headaches in pediatric populations are increasingly recognized as being associated with psychiatric disorders, predicting poor clinical outcomes. Despite global evidence, data from South Asia, particularly Bangladesh, remain sparse, limiting region‐specific insights into this comorbidity.

**Methods:**

This descriptive, cross‐sectional study was conducted in the Pediatric Neurology Departments of two tertiary care hospitals in Dhaka, Bangladesh, between July 2019 and March 2020. A total of 151 children and adolescents were assessed using the International Classification of Headache Disorders (ICHD‐III beta version) for headache classification and the validated Bangla version of the Development and Well‐Being Assessment (DAWBA) for psychiatric diagnoses.

**Results:**

Tension‐type headache was the most common type of headache (62.9%), followed by migraines (16.6%). Psychiatric co‐morbidities were identified in 39.7% of participants, with 13.2% presenting with multiple psychiatric disorders. Anxiety disorders (19.9%) and depressive disorders (12.6%) were the most prevalent. Children experiencing frequent headaches had significantly higher rates of psychiatric co‐morbidities (*p* = 0.020, 95% CI: 0.000–0.042). Logistic regression analysis revealed headache frequency as a minor but noteworthy predictor of psychiatric co‐morbidity (OR = 1.06, 95% CI: 1.016–1.093).

**Conclusion:**

This study highlights a high burden of psychiatric disorders among children and adolescents with headaches in Bangladesh, emphasizing the importance of psychiatric screening and multidisciplinary management approaches for pediatric headache. The findings provide valuable regional data and reinforce the need for pediatrician‐psychiatrist collaboration to improve outcomes.

## Graphical abstract

This study from Bangladesh found high psychiatric morbidity (39.7%) among 151 children and adolescents with headache. Anxiety disorders and depression was highest (19.9% and 12.6%). Higher frequency of headache was a mild predictor of psychiatric co‐morbidity. This highlights the importance of psychiatric screening in pediatric headache and pediatricians‐psychiatrists collaboration.

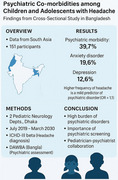

**Abbreviations**
BSMMU: Bangabandhu Sheikh Mujib Medical University
CDH: Chronic daily headache
DAWBA: Development and Well‐Being Assessment
DSM‐IV: Diagnostic and Statistical Manual of Mental Disorders
ICHD: International Classification for Headache Disorders
IPNA: Institute of Paediatric Neurodisorder and Autism
NINS: National Institute of Neuro Sciences
OCD: Obsessive‐compulsive disorder
PTSD: Post‐traumatic stress disorder
TTH: Tension‐type headache

## 1 Introduction

Headache is one of the most common neurological complaints among children and adolescents, often prompting visits to healthcare facilities. While not all headaches significantly impair quality of life, recurrent headaches can contribute to substantial physical, emotional, and social burdens, particularly when accompanied by psychiatric co‐morbidities. Despite their potential to exacerbate disease outcomes, the prevalence and types of psychiatric disorders in children and adolescents with headache remain poorly understood and under‐researched, particularly in low and middle‐income countries.

The International Headache Society (IHS) classifies headache into primary and secondary disorders. Primary headaches include migraine, tension‐type headache (TTH), trigeminal autonomic cephalalgias and others (Tepper 2013a). Epidemiological studies indicate that migraine and TTH are the most prevalent forms of headaches in pediatric population (Özge et al. 2011a).

Globally, in last decade the prevalence of headache in children has substantially increased (Albers et al. 2015). About 17.1% children in USA had frequent/severe headaches in 1 year (Lateef et al. 2009a). Among school‐going children, prevalence of headache was 10–20% which increased with age (Bellini et al. 2013a). In India, 66.4% school‐going children had headache with female preponderance (Malik et al. 2012). In Bangladesh, recurrent headache affected 17% school‐going children with migraine being most common type (55.3%) followed by TTH (19.6%). (Saha et al. 2017).

Research has consistently shown that headaches in children are associated with psychiatric disorders, including anxiety, depression, and behavioral issues, which may exacerbate the severity and frequency of headaches. In a systematic review, 22 out of 28 articles have found an association between headaches in children and psychological disorders, including both internalizing and externalizing symptoms (Polese et al. 2022). In Norway, recurrent headache was associated with symptoms of anxiety and depression among adolescents (Blaauw et al. 2014a). In another study, patients who had co‐occurring headaches reported significantly more depressive symptoms than those diagnosed solely with epilepsy (Pastorino et al. 2024). And behavioral disorder was higher among children with migraine than in general population (Pakalnis et al. 2005). In a representative study, one‐third of children and adolescents with primary headaches had emotional and behavioral problems (Just et al. 2003).

Despite these global findings, there is a lack of research exploring the psychiatric co‐morbidities associated with headaches in Bangladeshi children and adolescents. Understanding these correlations is crucial for improving the management and prognosis of pediatric headaches. This study aims to address this gap by exploring the proportion, types, and correlation of psychiatric disorders among children and adolescents with primary headaches in Bangladesh.

## 2 Methods

This was a descriptive type of cross‐sectional study conducted in two tertiary care centers in Dhaka: (a) the Institute of Pediatric Neurodisorder and Autism (IPNA), Bangabandhu Sheikh Mujib Medical University (BSMMU), Shahbagh, Dhaka and (b) Department of Pediatric Neurology, National Institute of Neuro Sciences (NINS), Sher‐E‐Bangla Nagar, Dhaka. These institutions were selected for their comprehensive services, nationwide patient catchment, and affordability, ensuring a diverse and representative sample.

Children and adolescents with headache (both new and old cases) aged between 5 and 17 years, were included after excluding major neurodevelopmental disorders (intellectual disability, autism spectrum disorder) and secondary headaches—due to trauma, tumor, infection or epilepsy. Sample was taken from outpatient departments of the study places between July 2019 and March 2020. The initial target sample size was 216, calculated using the formula *Z* ^2^ *pq*/*d* ^2^ with a prevalence (*p*) of 17% based on a prior study (Saha et al. 2017). Unfortunately, due to the COVID‐19 lockdown in March 2020, data collection was halted after 151 participants were enrolled using purposive sampling.

Data was collected by direct interview of the children/adolescent and their accompanying parents/primary care giver. The International Classification of Headache Disorders, 3rd edition beta version (ICHD‐3 beta) was used to classify headache types (Tepper 2013b). The term chronic daily headache (CDH) was used in the report though CDH is not an official diagnostic category in ICHD‐III. CDH is a widely used clinical umbrella term encompassing several chronic headache subtypes, including chronic tension‐type headache (CTTH), chronic migraine (CM), and new daily persistent headache (NDPH). This term is frequently employed in clinical practice and research to describe individuals experiencing headaches occurring on ≥15 days per month for at least three consecutive months (Laskar et al. 2019). CDH is a descriptive term rather than a formal ICHD‐III classification, ensuring alignment with standard headache classification systems.

Validated Bangla Version of Developments and Well–Being Assessment (DAWBA) (parent and self) was used to generate DSM‐IV psychiatric diagnosis (Mullick and Goodman 2005a). The DAWBA is a package of interviews, questionnaires and rating techniques designed to generate ICD‐10 and DSM‐IV or DSM‐5 psychiatric diagnoses on 2–17 year olds (Goodman n.d.). The DAWBA includes questions for 19 psychiatric disorder modules. The exact number of items varies per respondent, as the assessment employs skip rules to omit sections when initial responses indicate a low likelihood of certain diagnoses. The DAWBA covers the common emotional, behavioural and hyperactivity disorders. The parent interview takes around 50 min and the corresponding youth interview around 30 min. DAWBA—parent version was applied on caregiver and self‐version on the adolescents (11–17 years) by the researcher and four data collectors. Data collectors received hands‐on training from the lead researcher and the training aimed to promote consistency and minimize variability across assessments. The data collection was periodically supervised by the lead researcher. To ensure the reliability of psychiatric diagnoses, only a single certified rater finalized the diagnostic inferences. While DAWBA allows for DSM‐5 diagnoses, we opted to use DSM‐IV primarily because of the classification differences in ADHD. In DSM‐5, ADHD falls under neurodevelopmental disorders, whereas in DSM‐IV, it is categorized under behavioral disorders. Since our study excluded neurodevelopmental disorders, using DSM‐5 criteria would have created a conflict by including ADHD. Notably, apart from ADHD, the diagnostic criteria for other disorders remained largely unchanged between DSM‐IV and DSM‐5.

Informed consent was obtained from parents or caregivers, and assent was obtained from participants. Appropriate ethical clearance was secured from the Institutional Review Board. Data was analyzed using Statistical Package for Social Sciences (SPSS) version 23. Descriptive statistics, chi‐square tests, and logistic regression were applied to explore relationships between headache characteristics and psychiatric co‐morbidities. Mediation and moderation analyses were conducted using the PROCESS macro (version 4.2) in SPSS (Igartua and Hayes 2021).

## 3 Results

About 160 participants were approached, 9 were excluded for meeting exclusion criteria/not giving consent. Among the 151 respondents, 77 (51%) were from the National Institute of Neurosciences (NINS), and 74 (49%) were from Bangabandhu Sheikh Mujib Medical University (BSMMU). The gender distribution showed a slight female predominance, with 79 (52.3%) girls and 72 (47.7%) boys. Most participants, 86 (56.9%), resided in urban areas, while the remainder were from rural settings. The mean age of the respondents was 11.3 ± 2.5 years. A majority, 130 (86.1%), lived at home, while a small proportion resided in hostels. Regarding education, 126 (83.4%) children attended school, with most enrolled in mainstream schools (69.5%) and some in religious institutions (27.2%). The majority of participants were from middle‐income families (58.9%).

Tension‐type headache (TTH) was the most common type of headache, affecting 95 (62.9%) participants, followed by migraine in 25 (16.6%), and a combination of both migraine and TTH in 22 (14.6%) cases (Table 1). Age and gender distribution analysis showed that migraine and chronic daily headaches were more prevalent among girls, while TTH was more common among boys. However, these differences were not statistically significant. Gender‐specific analysis revealed that anxiety and behavioral disorders were more common in boys, while depressive disorders were more frequent in girls. Adolescents exhibited a significantly higher prevalence of “other problems” compared to younger children (*p* = 0.003) (Table 2).

**TABLE 1:** Age and gender distribution among respondents (*n* = 151) according to types of headaches.

	Sex				Age group					
	Boy		Girl		6–10 years		11–16 years		Total	
Type of headache	*n*	%	*n*	%	*n*	%	*n*	%	*n*	%
**Migraine**	8	11.1	17	21.5	11	17.7	14	15.7	25	16.6
**Tension type headache**	51	70.8	44	55.7	40	64.5	55	61.8	95	62.9
**Combined headache**	11	15.3	11	13.9	8	12.9	14	15.7	22	14.6
**Chronic daily headache**	2	2.8	7	8.9	3	4.8	6	6.7	9	6.0
**Total**	72	47.7	79	52.3	62	41.1	89	58.9	151	100

**TABLE 2:** Age and gender distribution and their relationship with psychiatric co‐morbidity (*n* = 151).

	Gender					Age group				
Psychiatric disorders	Boys		Girls			6–10 years		11–16 years		
	n	%	n	%	*p*	n	%	n	%	*p*
**Anxiety disorder**	16	22.2	9	11.4	0.083	11	17.7	14	15.7	0.825
**Depressive disorder**	7	9.7	12	15.2	0.338	6	9.7	13	14.6	0.459
**Behavioral disorder**	7	9.7	4	5.1	0.353	5	8.1	6	6.7	1.00
**Other problems**	8	11.1	12	15.2	0.483	2	3.2	18	20.2	0.003*

Frequency of headache (days per month) was much lower in migraine than in TTH and combined headache and the mean difference (5.2 ± 4.03 vs 15.9 ± 8.49) was significant using one way ANOVA (*p* < 0.001) (Table 3).

**TABLE 3:** Comparing means of frequency of headache days (in a month) between groups of headaches (*n* = 151).

Headache types	n	Mean	SD	SE	95% CI		F	*p*
					Lower	Upper		
**Migraine**	25	5.2	4.031	0.806	3.54	6.86		
**TTH**	95	15.94	8.397	0.861	14.23	17.65	24.978	0.000
**Combined headache**	22	19.27	8.697	1.854	15.42	23.13		

* Chronic Daily hedache was excluded from analysisof ANOVA.

Psychiatric co‐morbidities were identified in 60 (39.7%) respondents, with 20 (13.2%) experiencing more than one psychiatric problems. Anxiety disorders were the most prevalent, affecting 25 (19.9%) participants, followed by depressive disorders in 19 (12.6%) individuals (Table 4). Behavioral disorders were observed in 11 (7.3%) participants, while conditions grouped under “other problems” (including PTSD, OCD, and self‐harm) were present in 20 (13.3%) cases (Table 4). Anxiety disorders were higher than depressive disorder in all headache groups but behavioral and ‘other’ problems were more common among individuals with TTH (Table 5) but the difference was not significant

**TABLE 4:** Psychiatric disorders among respondents (*n* = 151).

Psychiatric disorder	n	%
Any psychiatric disorder	60	39.7
Any anxiety disorder	25^*^	19.87
Separation anxiety disorder	7	4.64
Specific phobia	11	7.28
Social phobia	6	3.97
Generalized Anxiety Disorder (GAD)	6	3.97
Depressive disorder	19	12.58
Behavioural disorders	11	7.28
Attention‐Deficit/Hyperactivity Disorder (ADHD)	1	0.66
Oppositional Defiant Disorder (ODD)	7	4.64
Conduct disorder	3	1.99
Other problems	20^**^	13.25
Self harm	6	3.97
Obsessive Compulsive Disorder (OCD)	4	2.65
Post‐Traumatic Stress Disorder (PTSD)	4	2.65
Others	8	5.3

* 5 respondents had more than one anxiety disorder.

** 2 respondents had co‐morbid ‘other problems’.

**TABLE 5:** Proportion of psychiatric diagnosis according to types of headaches (*n* = 151).

	Psychiatric diagnosis										
	Anxiety disorder		Depressive disorder		Behavioural disorder		Other problems				
Types of headache	*n*	%	*n*	%	*n*	%	*n*	%	*p*	*X^2^*	df
**Migraine**	6	24	3	12	0	0	2	8	0.08	6.61	3
**Tension type headache**	11	11.6	9	9.5	10	10.5	14	14.7	0.16	5.18	3
**Combined headache**	7	31.8	6	27.3	0	0	2	9.1	0.15	5.37	3
**Chronic daily headache**	1	11.1	1	11.1	1	11.1	2	22.2	0.63	1.74	3

**FIGURE 1:**
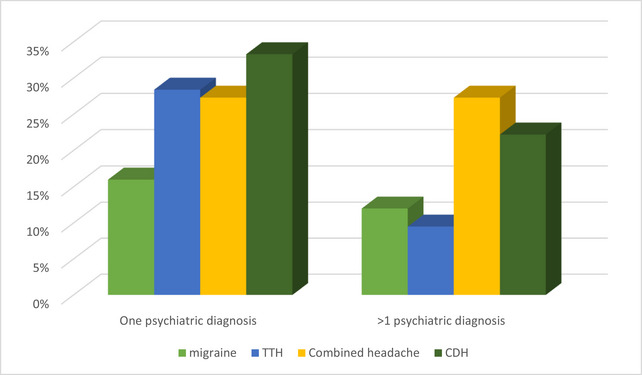
Number of psychiatric diagnosis according to headache types (*n* = 151).

Participants with chronic daily headaches (22.2%) and combined headaches (27.3%) were more likely to have multiple psychiatric co‐morbidities compared to other headache types. However, these differences were not statistically significant (Figure 1). Similarly, psychiatric co‐morbidities were more prevalent among individuals with combined and chronic daily headaches than those with migraines or TTH, but the difference was not significant (Table 6).

**TABLE 6:** Association of psychiatric co‐morbidity with type of headache (*n* = 151).

	Psychiatric co‐morbidity						
	Yes		No			95% CI	
Types of headache	*n*	%	*n*	%	*P*	Lower	Upper
**Migraine (*n* = 25)**	7	28	18	72			
**Tension type headache (*n* = 95)**	36	37.9	59	62.1	0.212	0.147	0.277
**Combined headache (*n* = 22)**	12	54.5	10	45.5			
**Chronic daily headache (*n* = 9)**	5	55.6	4	44.4			

X2 = 4.5, df = 3.

Children with more frequent headaches were significantly more likely to have psychiatric co‐morbidities (*p* = 0.020, 95% CI: 0.000–0.042) (Table 7). Logistic regression analysis revealed that higher headache frequency was a minor but significant predictor of psychiatric co‐morbidities (OR = 1.06, 95% CI: 1.016–1.093) (Table 8). Mediation analysis revealed that bootstrap confidence interval for the indirect effect of headache characteristics (total duration of headache and duration of individual episodes) were below zero (−0.003 to 0.005 and −0.018 to 0.020) suggesting that these factors did not mediate the association between frequency of headache and psychiatric morbidity. In moderation analysis, interaction effect of factors (age, gender and family income) were not statistically significant (*p* = 0.756, 0.202, 0.168) indicating that these factors did not moderate the effect of frequency of headache on psychiatric morbidity.

**TABLE 7:** Association of psychiatric co‐morbidity with frequency of headache (days in a month) (*n* = 151).

	Psychiatric co‐morbidity								
Days with headache in a month	Yes		No		Total			95% CI	
	*n*	%	*n*	%	*n*	%	*p*	Lower	Upper
**1–7 days**	6	10	22	24.2	28	18.5			
**8–20 days**	30	50	47	51.6	77	51	0.020	0.000	0.042
**>20 days**	24	24	22	24.2	46	30.5			
**Total**	60	100	91	100	151	100			

*X*2 = 6.9, df = 2.

**TABLE 8:** Binary Logistic regression with psychiatric co‐morbidity as dependent variable and frequency of headache per month (in days) and age, sex, family income as independent variables (*n* = 151).

Variables	B	S. E.	Wald	df	Sig.	Exp (B) [OR]	95%CI	
							Lower	Upper
**Age**	−0.014	0.069	0.041	1	0.839	0.986	0.862	1.129
**Frequency of headache (days per month)**	0.052	0.019	7.794	1	0.005	1.054	1.016	1.093

## 4 Discussion

This study aimed to explore the proportion and correlation of psychiatric disorders among children and adolescents with primary headaches, alongside exploring the relationship between headache types, frequency, and psychiatric co‐morbidities.

The mean age of participants (11.3 ± 2.5 years) and the female‐to‐male ratio (1.1:1 in 6–10 years and 1.3:1 in 11–16 years) are consistent with the previous research. A study in Bangladesh reported a mean age of 12.6 ± 1.08 years among Bangladeshi schoolchildren with headaches (Hoque et al. 2012), and Lateef et al. observed that headache prevalence becomes higher among girls post‐puberty (Lateef et al. 2009b). These trends likely reflect hormonal and psychosocial changes during adolescence, which may increase susceptibility to headaches among girls. However, our study found a less pronounced female preponderance compared to studies like Bellini et al. which reported a female‐to‐male ratio of 2.5:1 among adolescents (Bellini et al. 2013b). This discrepancy may be explained by the smaller sample size in our study, limiting its ability to reflect gender‐specific trends comprehensively.

Tension‐type headache (TTH) was the most common type of headache in our study, affecting 62.9% of participants, followed by migraine (16.6%) and combined headache (14.6%). These findings are consistent with national and international studies. Hoque et al. found TTH to be the most prevalent headache type (71.1%) followed by migraine (18.4%) among Bangladeshi children (Hoque et al. 2012), and a population‐based study in Italy reported similar distributions (migraine 28.6%, TTH 60.4%, and combined headache 14.3%; Maffioletti et al. 2015).

Our study revealed highly significant differences (*p* = 0.000) in headache frequency between TTH and migraine, with TTH patients experiencing headaches more frequently (15.9 ± 8.49 days per month) than those with migraine (5.2 ± 4.03 days per month). This aligns with the findings by Machnes‐Maayan et al. who reported similar trends (Machnes‐Maayan et al. 2014). Frequent headaches in TTH may exacerbate stress and lead to a vicious cycle, increasing susceptibility to psychiatric disorders. The chronic nature of TTH further underscores the importance of early intervention to prevent progression to chronic daily headache (CDH).

CDH was prevalent in our study population, particularly among girls and adolescents. Our findings are similar to a German study who reported higher rates of recurrent headaches among older girls (Kröner‐Herwig et al. 2007). About 55.6% of CDH children had psychiatric co‐morbidities that aligns with Ozge et al. who reported a prevalence of 64% among CDH patients (Özge et al. 2011a). This highlights the need for comprehensive management strategies that address both recurrent headache symptoms and underlying psychiatric conditions.

Psychiatric disorders were present in 39.7% of children and adolescents, significantly exceeding the general community prevalence of 15%–18% reported in Bangladeshi studies (Mullick and Goodman 2005b; Rabbani et al. 2009). Anxiety disorders were the most common psychiatric co‐morbidity, affecting 19.9% of participants, followed by depressive disorders (12.6%) and behavioral disorders (7.3%). These findings are consistent with another study that used similar instrument (DAWBA), and reported that anxiety was the most prevalent form of psychiatric disorder (68.8%) among children with headaches (Machnes‐Maayan et al. 2014). Also in Italy, significant higher internalizing problems (*p* = 0.023) was found in adolescents with headaches compared to the control group (Operto et al. 2018). The higher prevalence of psychiatric disorders may be attributed to the chronic and debilitating nature of headaches, which can significantly impact daily functioning and quality of life of children and adolescents. Frequent headaches may lead to increased stress, social isolation, and academic difficulties, all of which contribute to the development of psychiatric disorders.

Gender differences in psychiatric co‐morbidities were observed in our study, with anxiety and behavioral disorders more common among boys and depressive disorders more prevalent among girls. Although these differences were not statistically significant, they align with the findings from a population‐based study in Norway, who reported significantly higher rates of anxiety and depression among adolescent girls (Blaauw et al. 2014b). Hormonal changes, societal expectations, and coping mechanisms may contribute to these gender differences. However, the lack of significance in our study may be due to the small sample size, highlighting the need for larger studies to explore these trends further.

The association between headache type and psychiatric co‐morbidities revealed that TTH was more frequently associated with psychiatric disorders than migraine. This finding aligns with studies, which reported higher rates of psychiatric co‐morbidities among children with TTH (O'Brien and Slater 2016; Eidlitz‐Markus et al. 2017). The chronic and frequent nature of TTH may contribute to its stronger association with psychiatric disorders, as persistent pain can lead to emotional distress and maladaptive coping mechanisms. Combined headaches and CDH were associated with the highest rates of psychiatric co‐morbidities in our study, a finding supported by Antonaci et al., who reported that chronic headache patients were more likely to develop affective and anxiety disorders (Antonaci et al. 2011)

The significant association between headache frequency and psychiatric co‐morbidities observed in our study underscores the importance of addressing headache frequency in clinical practice. Research suggested that headache‐free intervals are vital for mental health and frequent headaches in adolescents increase the symptoms of depression and anxiety (Blaauw et al. 2014b). Logistic regression analysis in our study identified headache frequency as a mild predictor of psychiatric co‐morbidities (OR = 1.06, 95% CI: 1.016–1.093). This finding is consistent with prior research, which has emphasized the role of pediatric headache frequency in predicting mental health outcomes even in adulthood (Fearon and Hotopf 2001). Also in a systematic review, expressing negative emotions through anxiety and depression was found to be a predictor of persistent recurrent headache (Huguet et al. 2016). Conversely, many research findings indicate psychiatric disorders as a predictor of recurrent headache. A study revealed association between various preexisting mental disorders and the later development of frequent or severe headaches in general population worldwide (Bruffaerts et al. 2015). The bi‐directional relationship between chronic headaches and psychiatric disorders necessitates a multidisciplinary approach to management. Headaches were highly prevalent in children with mental health disorders in USA, and evaluation for coexisting depression and anxiety was recommended (Hommer et al. 2022). Similarly, in our study, the high prevalence of psychiatric disorders (39.7%) and its association with frequent headaches urges the need for integrating mental health evaluation for children with headache.

This study has several limitations. The final sample size (*n* = 151) was lower than the intended target (*n* = 216) due to COVID‐19, which may have reduced statistical power, particularly for detecting smaller effects or conducting subgroup analyses. But the sample size remains reasonably fit for descriptive study and the overall trends observed are still informative. The lack of a control group limits comparison with the general pediatric population, and the cross‐sectional design was insufficient to draw conclusion on causality between headache and psychiatric symptoms. It remains unclear whether psychiatric disorders are forecasters or consequences of recurrent headaches which highlights the need for longitudinal studies. As formal inter‐rater reliability was not assessed and data was self‐ or caregiver‐reported, this may introduce reporting bias. Genetic and epigenetic susceptibilities (such as familial patterns, shared genetic risk factors, and environmental stressors) were not assessed which could be a confounding variable in this study. Despite these limitations, the use of a validated instrument (DAWBA) and data from two reputable centers strengthen the validity of the findings. Future research should involve larger, randomized samples with control groups and longitudinal designs to clarify causal relationships.

Though the study was small, the findings advocate for routine screening for psychiatric disorders, particularly anxiety and depression in children and adolescents with frequent headaches. As psychiatric co‐morbidities have significant impact on headache frequency, severity, and overall prognosis, early identification and intervention may prevent chronicity and reduce long‐term functional impairment. Strengthening pediatric–psychiatric collaboration through liaison services can enhance timely referrals, promote shared decision‐making, and support comprehensive, multidisciplinary care. Public health efforts should also focus on increasing awareness among health care providers and training to better recognize and manage these overlapping conditions.

## 5 Conclusion

This study highlights the high prevalence of psychiatric disorders among children and adolescents with primary headaches, with anxiety and depressive disorders being the most common. Higher headache frequency was significantly associated with psychiatric co‐morbidities, underscoring the importance of addressing mental health concerns in the management of pediatric headaches. These findings stay as valuable regional data and emphasize the need for psychiatric assessments in recurrent pediatric headache. Integration of pediatric–psychiatric liaison services can ensure comprehensive care for headache in children and adolescents and improve their overall quality of life.

## Author Contributions

**Sifat E. Syed**: conceptualization, funding acquisition, writing – original draft, methodology, visualization, writing – review and editing, formal analysis, data curation. **Mohammad S. I. Mullick**: conceptualization, writing – review and editing, supervision

## Conflicts of Interest

The authors declare no conflicts of interest.

### Peer Review

The peer review history for this article is available at https://publons.com/publon/10.1002/brb3.70599

## Data Availability

The datasets generated and/or analyzed during the current study are available from the corresponding author on editor request. Any data shared will comply with institutional guidelines and ethical considerations to protect participant anonymity.
